# Association between *Rab31/*rs9965664 polymorphism and immunoglobulin therapy resistance in patients with Kawasaki disease

**DOI:** 10.3389/fcvm.2022.944508

**Published:** 2022-10-18

**Authors:** Hongyan Yu, Yueling Lin, Yufen Xu, Kaining Chen, Yishuai Wang, Lanyan Fu, Huazhong Zhou, Lei Pi, Di Che, Xiantao Qiu, Xiaoqiong Gu

**Affiliations:** ^1^Department of Clinical Biological Resource Bank, Guangzhou Institute of Pediatrics, Guangdong Provincial Key Laboratory of Research in Structural Birth Defect Disease, Guangzhou Women and Children's Medical Center, Guangzhou Medical University, Guangzhou, China; ^2^Department of Clinical Lab, Guangzhou Women and Children's Medical Center, Guangzhou Medical University, Guangzhou, China; ^3^Department of Blood Transfusion and Clinical Lab, Guangzhou Institute of Pediatrics, Guangzhou Women and Children's Medical Center, Guangzhou Medical University, Guangzhou, China

**Keywords:** Kawasaki disease, intravenous immunoglobulin, IVIG resistance, polymorphism, *Rab31/*rs9965664

## Abstract

**Background:**

Kawasaki disease (KD) is an acute febrile systemic vasculitis affecting infants and young children. A high dose of intravenous immunoglobulin (IVIG) is the first-line strategy for patients with KD to reduce persistent inflammation and the risk of coronary artery aneurysm (CAA) formation. Unfortunately, 10–20% of the patients showed no response to the treatment and were defined as resistant to IVIG. *Rab31* has been reported to regulate innate immunity in several human diseases. However, whether single nucleotide polymorphism (SNP) in *Rab31* gene could predispose to IVIG therapy response in KD was uncovered.

**Methods:**

*Rab31/*rs9965664 polymorphism was genotyped in 1,024 Chinese patients with KD through TaqMan assay. The odds ratios (ORs) and 95% confidence intervals (CIs) were calculated to assess the strength of association between *Rab31/*rs9965664 polymorphism and IVIG therapeutic effects.

**Results:**

Our results showed that *Rab31/*rs9965664 AA/GA genotype was significantly associated with an increased risk of IVIG resistance compared to GG genotype (GA vs. GG: *p* = 0.0249; AA vs. GG: *p* = 0.0016; AA/GA vs. GG: *p* = 0.0039; and AA vs. GG/GA: *p* = 0.0072). Moreover, the KD individuals carrying the rs9965664 A allele displayed lower Rab31 protein levels, and the expression level of Rab31 in the IVIG-resistant group was decreased significantly when compared to that observed in the response group. A mechanical study demonstrated that Rab31 modulated IVIG response through NLRP3 and p38 pathways.

**Conclusion:**

These results suggested that *Rab31/*rs9965664 polymorphism might be associated with an increased risk of IVIG resistance in southern Chinese patients with KD. The possible mechanism is that *Rab31* regulates the NLRP3 pathway negatively to inhibit IVIG response.

## Introduction

Kawasaki disease (KD) is an acute febrile systemic vasculitis that is usually diagnosed by a cluster of signs and symptoms (high fever, mucocutaneous inflammation, and cervical lymphadenopathy), and was first identified by Dr. Tomisaku Kawasaki (1). The KD is usually associated with multisystem disorders, while it prefers small- and medium-sized arteries, especially coronary arteries (2). Coronary aneurysms were observed in approximately 25% of the untreated patients (3–5), which makes KD the leading cause of acquired heart diseases in developed countries (5). Since the outbreak of the COVID-19 pandemic, several countries had reported multisystem inflammatory syndrome in children (MIS-C). The patient's clinical features were similar to KD (i.e., non-purulent conjunctivitis, polymorphic rash, mucosal changes, and swollen extremities) (6). In addition, Verdoni et al. pointed out that COVID-19 was associated with a high incidence of a severe form of KD (7). Therefore, currently, it is urgent to find an efficient clinical therapy for KD.

Intravenous immunoglobulin (IVIG) is a purified product that is obtained from the plasma of several thousands of donors (8). It is widely used for multiple autoimmune and inflammatory diseases, such as immune thrombocytopenia (ITP) and KD (9, 10). At present, a high dose of IVIG treatment remains the first-line strategy for patients with KD, as IVIG therapy can reduce persistent inflammation and the risk of coronary artery aneurysm (CAA) formation, which leads to sudden death (11). Unfortunately, 10–20% of the patients showed no response to the treatment and developed resistance to IVIG eventually (12). The IVIG-resistant patients had a higher risk of suffering from life-threatening complications like Kawasaki disease shock syndrome (KDSS) or KD macrophage activation syndrome (KD-MAS) (13). Understanding the mechanism of IVIG is critical for the clinical treatment of KD.

Human *Rabs* (Ras-related proteins in the brain) belong to the *Ras* family of small GTPases and have more than 60 members in the family (14). The function of *Rabs* depends on their molecular switch between the GTP ‘bound-on' form and the GDP ‘bound-off' form (14), and regulation of the special membrane trafficking in cell exocytose and endocytic progress (15–17). *Rab31* is also an important subgroup of the *Rabs* superfamily (18, 19) and is primarily localized on the Golgi complex and the organelle membrane originated from the Golgi (20). Along with membrane trafficking function, Rab31 recruited TBC1D2B to control the heterogeneous biogenesis of exosomes (21). Rab31 has also been reported to be essential for innate immunity. Yeo et al. found that Rab31 recruited signaling adaptor APPL2 during PI(3, 4)P2 to PI(3–5)P3 transition process in early phagocytosis and subsequently promoted Fc-gamma receptor (FcγR)-mediated phagocytosis *via* PI3K signaling pathway in macrophages (22).

Phagocytosis is vital for inflammatory reactions and autoimmune reactions for removing pathogens and tissue debris (23). More interestingly, the mechanism of IVIG action in immune thrombocytopenia and hemolytic anemia is by preventing phagocytosis of autoantibody-opsonized blood cells by competing for binding with FcγRs of macrophages in the spleen and live (24). It has been suggested that *Rab31-*modulated macrophage-associated phagocytosis may be critical for the IVIG effect, while there was a gap in *Rab31* function in KD or IVIG. To identify the function of *Rab31* in providing a response to IVIG therapy in patients with KD, we performed this study.

## Materials and methods

### Study population

A total of 1,024 patients with KD from Guangzhou Women and Children's Medical Center between January 2014 and December 2021 were enrolled in this study. All the patients recruited in this study were from the Han nationality of Southern China. Retrospective cross-referencing of the hospital database and the heart center echocardiography database confirmed the diagnosis and treatment of all participating patients with KD.

### Clinical diagnosis of KD

All individuals with KD were diagnosed by pediatricians based on the criteria of the American Heart Association (3, 25).

### Treatment response

Treatment response was determined as persistent or recrudescent fever (temperature ≥38.0°C, measured axilla or orally) for at least 36 h but no longer than 7 days after the completion of the first IVIG infusion (2 g/kg) (3).

### Polymorphism genotyping and DNA extraction

Peripheral blood was collected from patients with KD. Genomic DNA was extracted with the TIANamp Blood DNA Kit (DP318, TIANGEN Biotech, Beijing) followed by the guidance of the manufacturer's instructions. Specific fluorescent allele probes for rs9965664 were purchased from ABI (Thermo Fisher Scientific, United States). PCR was performed in 384-well plates with an ABI-Q6 Sequence Detection System machine (Thermo Fisher Scientific).

The genotyping of the SNP was conducted using the TaqMan SNP genotyping assay. Laboratory technicians were blind to the sample information, including the identities of the replicate aliquots. About 10% of the samples from both groups were arbitrarily chosen to repeat the genotyping results. A concordance rate of 100% was obtained.

### Western blots

Total PBMC protein was extracted from cells using cell lysis buffer containing PMSF (Beyotime). The samples were separated by 12.5% SDS-polyacrylamide gel electrophoresis, followed by transferring to the PVDF membrane. After blocking in PBS containing 5% bovine serum albumin, the membrane was incubated with primary Rab31 antibody (1:1,000; 16182-1-AP, Proteintech), NLRP3 antibody (1:1,000; 15101,Cell Signaling Technology), p-P38 antibody (1:1,000; ARG51850, Arigo), or beta-actin antibody (1:5,000; 380624, Zen BioScience) at 4°C overnight, followed by incubation with a peroxidase-linked secondary antibody (Anti-rabbit IgG, HRP-linked Antibody, 1:5,000; 7074S, Cell Signaling Technology; or Anti-mouse IgG, HRP-linked Antibody, 1:2,000; 7076S, Cell Signaling Technology) at room temperature for 1 h. The signals were detected by X-ray film after incubating with Western blotting Luminol reagent (GE Health care). All images were analyzed with ImageJ software.

### Statistical analysis

A statistical analysis of this study was performed by using SAS software (version 9.4; SAS Institute, Cary, NC). Pearson's Chi-square test was used to evaluate the significant differences between IVIG response and IVIG-resistant cases in the distribution of demographic variables and genotype frequency. Odds ratios (ORs) and 95% confidence intervals (CIs) were calculated by logistic regression analysis for measuring the association between the *Rab31*/rs9965664 polymorphism and the risk of IVIG treatment resistance in patients with KD. The statistical graphs of relative protein expression (fold change) were drawn with GraphPad Prism 8. The *p*-values < 0.05 were regarded as statistically significant.

## Results

### Characteristics of patients with Kawasaki disease

A total of 1,024 patients with KD were enrolled in this study (Table 1). The characteristic distribution of 817 IVIG therapy response KD patients and 207 IVIG therapy resistant KD patients are presented in Table 1. The percentage of the IVIG-resistant population was 20.2%, which was consistent with the previous study (13). The average age of the IVIG response group was 25.68 ± 20.65 months (range 1–131 months), and it was 27.69 ± 22.68 months (range 2–132 months) for the IVIG-resistant group. About 62.55% of patients with KD who responded to IVIG therapy were men, and the proportion of male patients who were resistant to IVIG therapy was 67.63%. The proportions of females were 37.45 and 32.37%. There were no significant differences in age (*p* = 0.189) and gender (*p* = 0.172) between the IVIG response group and the resistant group.

**Table 1:** Characteristics distribution in IVIG therapy resistant group and response group of KD patients.

**Variables**	**IVIG resistance (*****n*** = **207)**		**IVIG response (*****n*** = **817)**		***p* ^a^**
	**No**.	**%**	**No**.	**%**	
Total	207	100	817	100	
Age range, month	2–132		1–131		
Mean ± SD	27.69 ± 22.68		25.68 ± 20.65		
≤ 60	191	92.27	774	94.74	0.189
>60	16	7.73	43	5.26	
Gender
Male	140	67.63	511	62.55	0.172
Female	67	32.37	306	37.45	

^*a*^Two-sided χ^2^test for distributions between KD patients in IVIG therapy resistance group and response group.

The incidence of coronary artery complications was explored (Table 2). About 54.59% of patients from the IVIG-resistant group suffered coronary artery lesions, while the percentage decreased to 36.23% in the IVIG response group.

**Table 2:** The incidence of coronary complication in IVIG therapy resistant group and response group of KD patients.

**Coronary artery outcomes**	**IVIG resistance**	**IVIG response**
CAL^a^	113 (54.59)	296 (36.23)
NCAL^b^	94 (45.41)	521 (63.77)
Total	207	817

^a^Coronary artery lesions.

^b^Coronary without artery aneurysm and dilatation.

### Analysis of the associations between *Rab31*/rs9965664 polymorphism and IVIG resistance

The distribution of genotype frequency of *Rab31*/rs9965664 polymorphism in the KD IVIG-resistant group and response group is described in Table 3. To explore the association between *Rab31*/rs9965664 polymorphism and the risk for IVIG therapy resistance, we performed a χ2 test analysis. We found that *Rab31*/rs9965664polymorphism was significantly associated with increased IVIG therapy resistance risk in patients with KD (GA vs. GG: adjusted OR = 1.45, 95% confidence interval (CI) = 1.05–2.00, *p* = 0.025; AA vs. GG: adjusted OR = 2.46, 95% confidence interval (CI) =1.41–4.30, *p* = 0.002; AA/GA vs. GG: adjusted OR =1.57, 95% CI = 1.16–2.14, *p* = 0.004; AA vs. GA/GG: adjusted OR = 2.09, 95% confidence interval (CI) = 1.22–3.57, *p* = 0.007). The results indicated that patients with AA/GA genotype had a higher risk of suffering resistance to IVIG therapy compared to the patients with GG genotype, suggesting the resistive effect of this SNP against IVIG therapy.

**Table 3:** Genotype distribution frequency of *RAB31*/ rs9965664 polymorphism in KD patients with IVIG resistance and response therapy.

**Genotype**	**IVIG resistance (*N* = 207)**	**IVIG response (*N* = 817)**	***p*-value^a^**	**OR (95% CI)**	***p*-value**	**Adjusted OR (95% CI)**	***p*-value^b^**
GG	94 (45.41)	463 (56.67)	0.0033	1		1	
GA	91 (43.96)	309 (37.82)		1.451 (1.05–2.00)	0.0237	1.45 (1.05–2.00)	0.0249
AA	22 (10.63)	45 (5.51)		2.408 (1.38–4.20)	0.0019	2.46 (1.41–4.30)	0.0016
Additive				1.51 (1.19–1.92)	0.0007	1.52 (1.20–1.93)	0.0006
Dominant	113 (54.59)	354 (43.33)	0.0037	1.57 (1.16–2.14)	0.0038	1.57 (1.16–2.14)	0.0039
Recessive	185 (89.37)	772 (94.49)	0.012	2.04 (1.20–3.48)	0.0089	2.09 (1.22–3.57)	0.0072

^a^Two-sided χ2 test for distributions between KD patients in IVIG therapy resistance group and response group. ^b^Adjusted for age and gender status in logistic regress models.

### Stratification analysis

We further explored the association between *Rab31*/rs9965664 polymorphism and the risk effect of IVIG resistance in certain subgroups classified by age and gender (Table 4). Compared with the rs9965664/GG genotype, the risk effect of rs4594236 AA/GA genotype was more prominent in male patients (adjust OR = 1.86, 95% CI = 1.27–2.71, *p* = 0.001), especially aged less than 60 months (adjust OR = 1.56, 95% CI = 1.13–2.15, *p* = 0.006).

**Table 4:** Stratification analysis of *RAB31*/rs9965664 polymorphism in IVIG therapy resistant group and response group of KD patients.

**Variables**	**rs9965664 (IVIG resistance/ IVIG response)**		***p*-value^a^**	**OR (95% CI)**	***p*-value**	**Adjusted OR (95% CI)**	**Adjusted *p*-value^b^**
	**GG**	**GA/AA**					
Age, months
≤ 60	87/439	104/335	0.0056	1.57 (1.14–2.15)	0.0057	1.56 (1.13–2.15)	0.0063
>60	7/24	9/19	0.4093	1.62 (0.51–5.16)	0.4113	1.65 (0.52–5.29)	0.3975
Gender
Male	58/290	82/221	0.0013	1.86 (1.27–2.71)	0.0014	1.86 (1.27–2.71)	0.0014
Female	36/173	31/133	0.6757	1.12 (0.66–1.90)	0.6754	1.14 (0.67–1.94)	0.6366

^a^Two-sided χ2 test for distributions between Kawasaki disease patients in IVIG therapy resistance group and response group.

^b^Adjusted for gender/age status in logistic regress models.

### *Rab31* protein expression level decreased in PBMC of IVIG-resistant KD patients

**Figure 1:**
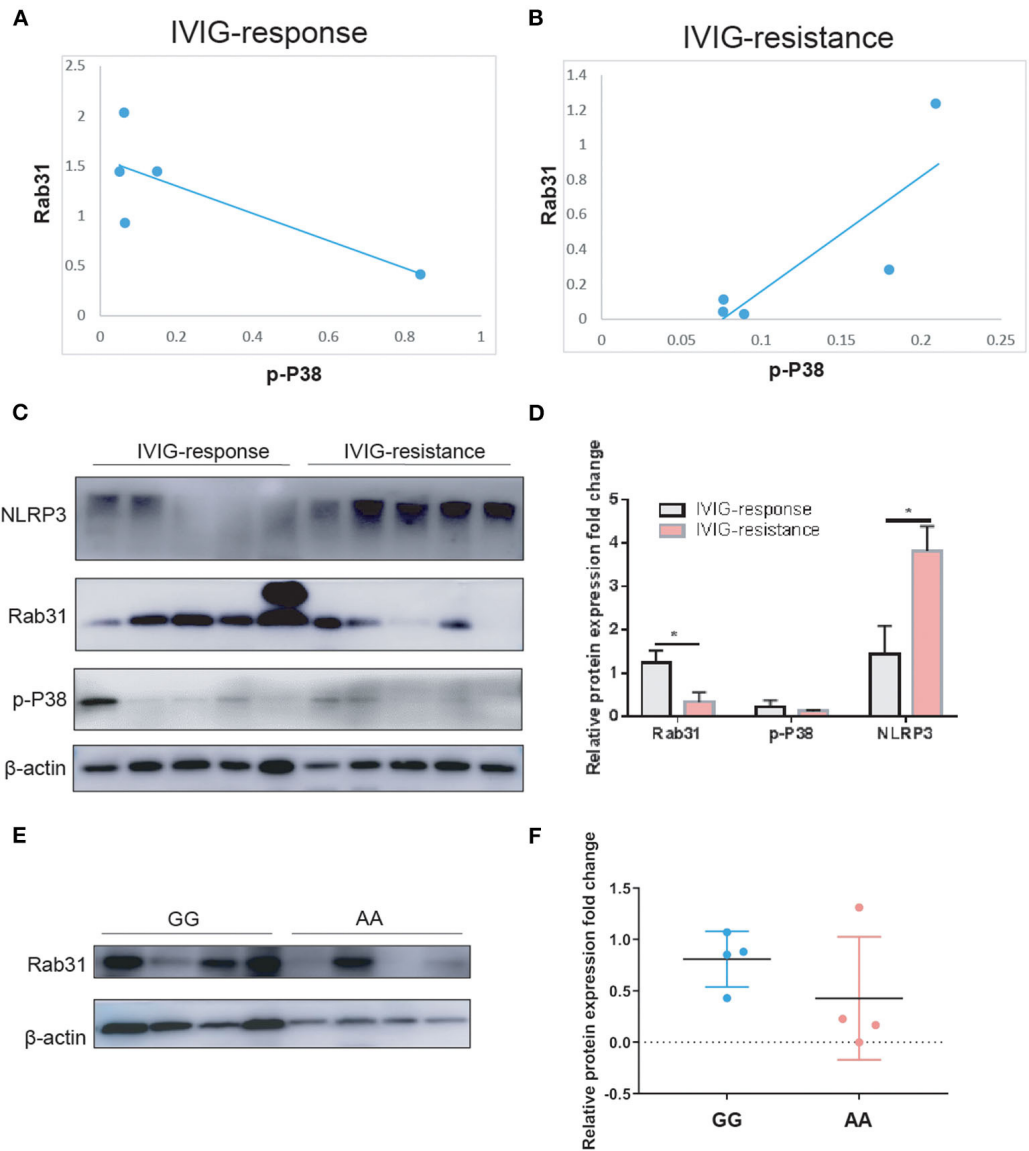
**(A)** Scatter diagrams for the correlation analysis of p-P38 and Rab31 expression in the IVIG response group. **(B)** Scatter diagrams for the correlation analysis of p-P38 and Rab31 expression in the IVIG-resistant group. **(C,D)** Representative Western blots and quantitative results of Rab31, p-P38, and NLRP3 protein expression in PBMC of patients with KD. **(E,F)** Representative Western blots and quantitative results of Rab31 protein expression in PBMC of different Rab31 genotypes of patients with KD.

To assess the role of Rab31 in the IVIG effect in patients with KD, we performed WB in the PBMCs of 10 patients with KD (five IVIG response and five IVIG resistance). The results showed that the protein expression of Rab31 in the IVIG-resistant group decreased significantly when compared to that in the response group (Figure 1). Rs9965664 of *Rab31* was located on the intron area of the 18th chromosome, and it is well known that introns can affect gene expression in many species in a variety of ways (26). To test whether rs9965664 could affect Rab31 protein expression, we measured Rab31 expression levels among different genotypes of rs996566, and the results showed that the individuals carrying the rs9965664 A allele displayed lower Rab31 protein levels in the PBMC, consistent with our IVIG results (Figure 1). We also explored the possible downstream pathway of *Rab31*, and found that the NLRP3 pathway was increased significantly in the IVIG-resistant group. More interestingly, there were no differences in the phosphorylation p38 levels between the IVIG response and resistant groups, while the protein level of Rab31 was positively associated with the p-P38 level in the IVIG-resistant group and negatively associated in the IVIG response group. In summary, the KD patient with rs9965664 A allele tends to have a lower Rab31 protein expression level, and decreased Rab31 may inhibit IVIG response through NLRP3 and p38 pathways.

## Discussion

To date, Rabs proteins have been implicated directly or indirectly in many human diseases (19). Knockdown of Rab5 inhibits early trafficking and fusion of endosomes, subsequently blocking the internalization of VEGF-induced vascular endothelial growth factor receptor 2 (VEGFR2), which is essential for endothelial cell growth and angiogenesis (27). *Rab31* is also a member of the Rab5 family proteins (18, 19), and has been reported to be associated with the malignant behavior of breast cancer, ovarian cancer, and glioblastoma (28–30). However, the *Rab31* gene is not known to be correlated with KD or IVIG therapy yet. An association of IVIG therapy effect with genetic variants of *Rab31* was not found in any population. Through genomic research, we examined the association between *Rab31* gene polymorphism and IVIG therapy in KD in a Chinese population.

In this study, we provided evidence for the role of *Rab31*/rs9965664 polymorphism in IVIG resistance to KD and for the first time reported that *Rab31*/rs9965664 polymorphism could predispose to IVIG resistance in Southern Chinese children with KD. To our knowledge, this is the first study to explore the association between *Rab31* gene SNP rs9965664 and IVIG therapy effects in KD.

Next, we tested the protein expression level of Rab31in the PBMC of 10 patients with KD (five IVIG response and five IVIG resistance), and the results showed that the Rab31 protein expression was decreased in the IVIG-resistant group when compared to the response group (Figure 1). This finding suggests that the transcriptional downregulation of Rab31 is involved in IVIG resistance. Keeping with this result, KD patients with A allele of rs9965664 genotype tend to have lower Rab31 protein expression levels, which was consistent with the previous results that KD patients with AA/GA genotype had a higher risk of suffering IVIG therapy resistance compared to the patients with GG genotype, while the mechanism needs to be explored in the future.

Intravenous immunoglobulin is the effective treatment for KD at present, while the molecular basis of IVIG action is still under investigation, but the proposed possible mechanisms are as follows: saturation of FcγR of macrophages to inhibit the immune cell from being over-activated, neutralization of infectious antigens and super antigens, and neutralization of pathogenic auto-antibodies (2, 31). Interestingly, Rab31 recruited macrophage signaling adaptor APPL2 to modulate macrophage phagocytosis through the regulation of phagocytic cup formation. Furthermore, APPL2 increased Akt and decreased p38 mitogen-activated protein kinase during FcγR activation (22). Our preliminary data showed that the rab31 protein expression level was decreased in the IVIG-resistant group, and the phosphorylation p38 level showed no differences between the two groups. However, within the IVIG response group, the Rab31 protein expression level tends to be negatively associated with the phosphorylated p38 level, which is in line with a previous study. However, the association was opposite in the IVIG-resistant group. In addition, the expression level of NLRP3 was increased in the IVIG-resistant group. Thus, we hypothesized that the lower Rab31 expression inhibited FcγR-dependent macrophage phagocytosis *via* increasing phosphorylated p38 level and activating the NLRP3 pathway to damage the response to IVIG therapy.

In summary, we identified that *Rab31*/rs9965664, which is a new locus, is associated with IVIG resistance in KD. Future studies are required to explore its functions in relation to the mechanism by which the rs9965664 genotype decreased Rab31 expression and increased IVIG resistance.

However, our study still has some limitations. First, the enrolled patients of this study were from the southern Chinese population, and the conclusion needs multi-center subjects from other geographic populations to support and evaluate the applicability of the findings to other ethnic groups. Second, only one functional SNP in the *Rab31* gene was included, more specifically functional *Rab31* SNPs need to be investigated in the future. Last but not least, the exact biological mechanism of *Rab31* in IVIG resistance of KD is worthy of further investigation.

## Data availability statement

The raw data supporting the conclusions of this article will be available on reasonable request.

## Ethics statement

The studies involving human participants were reviewed and approved by the Ethics Review Committee of Guangzhou Women and Children Medical Center (2014073009, 2018052702, and 2021093A01). Written informed consent to participate in this study was provided by the participants or their legal guardian/next of kin.

## Author contributions

Conceptualization: HY and XG. Methodology: YL and HY. Formal analysis: KC and YX. Investigation: HZ and DC. Data curation: YX, LF, and LP. Original draft preparation: HY, YL, and YX. Reviewing and editing: HY, YL, XQ, and XG. All authors read, reviewed, and approved the final manuscript.

## Funding

This study was funded by the Guangdong Natural Science Fund, China (Grant Nos. 2019A1515012061, 2021A1515011207, and 2022A1515012558), the Guangzhou Science and Technology Program Project, China (Grant Nos. 201904010486 and 202102010197), the Guangzhou Institute of Pediatrics/Guangzhou Women and Children's Medical Center Fund, China (Grant Nos. GCP-2019-003, GCP-2019-006, and YIP-2019-050), and Postdoctoral Research Initiation Fund from Guangzhou Institute of Pediatrics, Guangzhou Women and Children's Medical Center (Grant No. 3001162).

## Conflict of interest

The authors declare that the research was conducted in the absence of any commercial or financial relationships that could be construed as a potential conflict of interest.

## Publisher's note

All claims expressed in this article are solely those of the authors and do not necessarily represent those of their affiliated organizations, or those of the publisher, the editors and the reviewers. Any product that may be evaluated in this article, or claim that may be made by its manufacturer, is not guaranteed or endorsed by the publisher.
